# Mapping Sub‐National Respiratory Virus Circulation in Cambodia Using Metatranscriptomic Sequencing: A Multi‐Center Hospital‐Based Surveillance Study

**DOI:** 10.1111/irv.70306

**Published:** 2026-08-19

**Authors:** Christina Yek, Jessalyn Sebastian, Sophana Chea, Sreyngim Lay, Mengheng Oum, Lyhourng Long, Sreytouch Chea, Andrea R. Pacheco, Meera Barochia, Piseth Ly, Sokna Ly, Ratanak Sath, Daniel M. Parker, Volodymyr M. Minin, Matthew Chung, Elodie Ghedin, Fabiano Oliveira, Jessica E. Manning, Kimsreng Lean, Chanty Ny, Viseth Long, Kimhor Leang, Virak Yim, Kry Hok, Rithea Leang, Rekol Huy, Savuth Chin, Darapheak Chau, Heng Seng, Sovann Ly, Chanthap Lon

**Affiliations:** ^1^ International Center of Excellence in Research Cambodia Phnom Penh Cambodia; ^2^ Laboratory of Clinical Immunology and Microbiology National Institute of Allergy and Infectious Diseases Maryland USA; ^3^ Department of Statistics University of California Irvine California USA; ^4^ Faculty of Pharmacy University of Health Sciences Phnom Penh Cambodia; ^5^ Department of Population Health and Disease Prevention University of California Irvine California USA; ^6^ Laboratory of Parasitic Diseases National Institute of Allergy and Infectious Diseases Maryland USA; ^7^ Sanofi Pasteur Pennsylvania USA; ^8^ National Pediatric Hospital Phnom Penh Cambodia; ^9^ Preah Kossomak Hospital Phnom Penh Cambodia; ^10^ Kampong Speu District Referral Hospital Kampong Speu Cambodia; ^11^ National Center for Parasitology, Entomology, and Malaria Control, Ministry of Health Phnom Penh Cambodia; ^12^ National Institute of Public Health, Ministry of Health Phnom Penh Cambodia; ^13^ Communicable Disease Control Department, Ministry of Health Phnom Penh Cambodia

**Keywords:** biosurveillance, Cambodia, influenza (human), metagenomics, respiratory tract infections

## Abstract

**Background:**

Genomic surveillance can guide early detection of and response to emerging epidemics. Metatranscriptomic sequencing was used to investigate sub‐national respiratory virus circulation in Cambodia from 2020 to 2023.

**Methods:**

Nasopharyngeal swabs were collected from individuals aged 2 months to 65 years with influenza‐like illness in four Cambodian hospitals. Metatranscriptomic data were generated by short‐read RNA sequencing. Bernoulli space–time scan statistics were used to identify temporal virus clusters. Bayesian inference of phylogenetic trees was used to compute divergence times for temporally clustered, highly represented viruses (influenza A/H3N2 and B, *Betacoronavirus 1*, respiratory syncytial virus [RSV] A and B), and publicly available global influenza virus genomes.

**Results:**

Of 1093 individuals, 499 (45.7%) had detectable respiratory viruses belonging to 68 distinct species. Moderate (*N* > 20) discrete time‐clusters were noted of RSV‐A (37 cases), *Betacoronavirus 1* (21 cases), RSV‐B (22 cases), and A/H3N2 (30 cases). The posterior median of time to most recent common ancestor ranged from 0.71 years (95% HPD 0.38–1.10) for *Betacoronavirus 1* and 1.31 years (95% HPD 0.60–3.20) for A/H3N2, to 2.75 years (1.82–4.26) for RSV‐A and 4.79 years (2.39–7.74) for RSV‐B. A/H3N2 and influenza B virus genomes mapped to clades 3C.2a1b.2a.2a and Victoria 1A.3a.2, respectively, and inter‐mixed with concurrent global strains.

**Conclusions:**

Multiple respiratory viruses circulated at a sub‐national level in Cambodia from 2020 to 2023 despite pandemic disruptions. Influenza virus population diversity decreased during the height of lockdown but recovered in mid‐2022. Re‐emerging influenza strains were distinct from historically circulating strains and clustered with contemporaneous global variants, suggesting multiple external introductions.

## 1 Introduction

Following the introduction of SARS‐CoV‐2 in 2020, unusual fluctuations were described in the epidemiology of other respiratory viruses globally [1]. These changes were largely attributed to disrupted virus transmission from use of non‐pharmaceutical interventions and near‐cessation of global travel, although changes in host susceptibility, health‐seeking behavior, and testing practices may also have contributed [2].

The most dramatic changes were noted shortly after March 2020, when circulation of many non‐SARS‐CoV‐2 respiratory viruses dropped to a fraction of pre‐pandemic levels. The subsequent mid‐2020 Southern Hemisphere and 2020–2021 Northern Hemisphere winter seasons saw minimal influenza and RSV circulation [1]. Influenza cases remained largely suppressed through late 2021 [3, 4, 5, 6] apart from scattered off‐ [7] and early‐cycle [2] outbreaks. RSV activity returned more quickly with season starts mid‐2021 [4, 6, 8]. By 2022, many countries reported returning circulation of both RSV and influenza amidst gradual easing of social mobility restrictions. Genomic studies at this point revealed transmission bottlenecks in both virus populations with monophyletic resurgences, variably due to new population introductions [4, 9] or cryptically circulating lineages [8], before return of pre‐pandemic diversity. In a notable exception, the influenza B/Yamagata lineage failed to re‐emerge; its possible extinction was attributed to a combination of minimal antigenic evolution, transmission chain disruptions, and a shrinking susceptible host pool [10]. Global impacts of the pandemic on the dynamics and evolution of other common respiratory viruses such as rhino/enteroviruses, human parainfluenzaviruses, human metapneumovirus, and adenoviruses were less visible in the absence of widespread surveillance [11, 12].

The COVID‐19 pandemic triggered a rapid expansion of global laboratory and clinical infrastructure supporting respiratory virus surveillance. In Cambodia, a low‐middle‐income country (LMIC) in Southeast Asia, active sentinel surveillance for influenza‐like illness (ILI) and severe acute respiratory infection (SARI) was already in existence in 2009 [13, 14]. These networks were leveraged during the COVID‐19 pandemic to capture SARS‐CoV‐2 cases while simultaneously continuing to monitor ILI and SARI activity. In 2020, ILI surveillance captured a smaller‐than‐usual influenza season that included an A/H3N2 outbreak at a single pagoda [15]. In 2021, testing of ILI and SARI samples revealed invasion of alpha and delta SARS‐CoV‐2 variants associated with COVID‐19 case surges even amidst stringent mobility restrictions and widespread vaccine adoption [16].

In this study, we applied metatranscriptomic sequencing to detect and characterize known respiratory viruses in nasopharyngeal swabs from participants presenting with ILI to four tertiary hospitals in Cambodia between December 2020 and March 2023. We examine the data to report local respiratory virus epidemiology following the introduction of SARS‐CoV‐2.

## 2 Methods

### 2.1 Clinical Protocol and Enrollment

Participants aged 2 months to 65 years with ILI, defined as a documented temperature > 38°C within 24 h and concurrent respiratory symptoms (cough, coryza, or dyspnea) were enrolled at emergency departments and urgent care clinics at four tertiary referral hospitals in Cambodia. A single nasopharyngeal (NP) swab was collected by placing a swab in either naris for a 360° rotation. Participant demographics (age, sex, occupation, home location [village centroid], travel history) and clinical data (vital signs, other acute symptoms, basic laboratory results, co‐morbid disease) were collected using a standardized case report form (Table S1). The study protocol was approved by the National Institutes of Health Institutional Review Board and the Cambodian National Ethics Committee for Human Research and registered on clinicaltrials.gov (NCT04034264). Study reporting was consistent and conformed to the standards of the Strengthening the Reporting of Observational Studies in Epidemiology (STROBE) reporting guidelines for observational studies.

### 2.2 Sample Processing and Sequencing

NP swabs were stored in nucleic acid preservative (2 mL DNA/RNA Shield, Zymo Research) and transported to a central laboratory in Phnom Penh, Cambodia, where they were subjected to total RNA extraction (Quick‐RNA MicroPrep Kit, Zymo Research) and stored at −80°C. RNA libraries were prepared using the NEBNext Ultra II RNA Library Prep Kit (New England BioLabs). The full protocol is available at protocols.io (https://doi.org/10.17504/protocols.io.36wgq2j9ogk5/v1). Briefly, extracted RNA was mixed with random primers, reaction buffer, ERCC RNA spike‐in mix (Invitrogen), and QIAseq FastSelect rRNA (Qiagen) to remove human rRNA, and heat sheared in a thermocycler. cDNA was synthesized using NEBNext First Strand Synthesis Enzyme for first‐strand synthesis and SS enzyme mix and SS reaction buffer for second‐strand synthesis, and end prep performed using Ultra II End Prep reaction buffer and enzyme mix, according to manufacturer's instructions. Adapter ligation was performed using NEBNext Ultra II ligation master mix, NEBNext ligation enhancer, and NEBNext Adapters for Illumina (New England BioLabs). Digestion and barcoding were performed using USER enzyme (New England BioLabs), NEBNext Ultra II Q5 master mix, and NEBNext universal and index primers for Illumina (New England BioLabs). Purification was performed after second‐strand synthesis, adapter ligation, and barcoding using magnetic beads. Libraries were quantified using a TapeStation 4150 (Agilent Technologies) before and after pooling. Prepared RNA libraries were loaded on a NextSeq2000 P2 flow cell (Illumina) for 150 bp paired‐end read sequencing generation of 4–8 M paired reads per sample. Fastq files were deposited in NCBI's Sequence Read Archive under BioProject PRJNA681566 (accessions in Table S1).

### 2.3 Read Filtering and Reference‐Guided Assembly

Raw fastq files were processed in CZID (czid.org, Illumina mNGS pipeline v7.1). The pipeline removes adapters (Trimmomatic), performs host read subtraction (STAR and Bowtie2; human genome reference: GCF_000001405.26), removes low‐quality and duplicate reads (PriceSeq), and aligns remaining reads to the NCBI NT and NR databases (minimap2 and Diamond, respectively). Read reports were filtered for species that corresponded to known pathogenic viruses (https://czid.org/pathogen_list), constituted ≥ 10 NT reads per million, and for which NT reads were not present in any water controls. Species identity was confirmed by BLAST against NT and selecting only species with alignment scores ≥ 200. Consensus sequences were generated for hits with ≥ 90% genome (or segment, for segmented viruses) identity with a reference accession and ≥ 10× coverage depth using CZID's Viral Consensus Genome module (Table S2). Clades for RSV‐A, RSV‐B, influenza A, influenza B, and SARS‐CoV‐2 consensus sequences were identified with Nextclade v3.11.0 (https://clades.nextstrain.org).

### 2.4 Temporal Analyses of Lockdown Stringency and ILI Cases

The Oxford Stringency Index (OSI) is a composite measure of national and subnational policy measures including mobility restrictions implemented to reduce community transmission of SARS‐CoV‐2 during the global pandemic [17]. OSI data were obtained from Our World in Data/OxCGRT (accessed June 7, 2024). The relationship between ILI cases and OSI was explored by examining cross‐correlations between case counts grouped by month, monthly aggregate hospital admissions (for Kampong Speu, National Pediatric, and Preah Kossomak hospitals; data from National Maternal and Child Health Center were not available), and monthly mean OSI lagged by 1‐month increments, with between 0 and 4 lags using the R stats package (v4.3.1). Negative binomial regression models were constructed using the MASS package (v7.3.60.0.1 [18]) and fit to monthly ILI cases with the lagged and non‐lagged OSI and hospital admissions as predictors. Candidate OSI lags were examined using cross‐correlation functions and compared by Akaike information criterion. The final model had no lags and took the following construction: glm.nb (enrollment ~ OSI + admissions). Adjusted predicted values from the final model were plotted using the ggeffects package (v2.3.0 [19]).

### 2.5 Identification of Respiratory Virus Spatiotemporal Clusters

Discrete temporal and spatiotemporal clusters of respiratory virus cases were identified using SaTScan (v10.1.2 [20]). Detected viruses were grouped at the species level, except *Alphainfluenzavirus influenzae*, which was further grouped by H/N segment assignments (H3N2 and H1N1) and RSV which was grouped by strain (RSV‐A and RSV‐B); viruses with > 10 occurrences were selected for cluster analysis. For each virus of interest, cases were defined as detections of the virus and controls as all other ILI cases, including those without a detected viral pathogen. Retrospective space–time analysis was performed using a Bernoulli probability model with maximum temporal and spatial size set at 25% of the at‐risk population, enabling calling of pure temporal clusters (spatial size 100%), minimum cluster size of 5, and time precision in days. Time or space–time windows with a higher‐than‐expected proportion of the virus of interest were identified using 999 Monte Carlo replications and a minimum Monte Carlo *p* value of 0.01 specified for statistically supported clusters. For each viral species, pairwise geographic (linear distance between cases, with case location represented by village centroid [km]), genetic (number of small nucleotide polymorphisms [SNPs]), and temporal (time between sample collection [days]) distances were calculated for all pairs of samples with consensus genomes. Distance matrices were compared using Mantel tests with Spearman rank correlation and 9999 permutations for each viral species using the vegan package (v2.7.1) [21].

### 2.6 Public Influenza Virus Case and Sequence Data

Global influenza epidemiological data from January 1, 2020, to December 31, 2022, were obtained from all WHO regions through the Global Influenza Surveillance and Response System (GISRS; https://www.who.int/initiatives/global‐influenza‐surveillance‐and‐response‐system, accessed May 28, 2025). All complete sequences with complete metadata fields for collection date and country for A/H3N2 and influenza B HA isolated from human sources in Vietnam, Thailand, Laos, and Cambodia (part of the Greater Mekong Subregion [GMS]) were downloaded from the Global Initiative on Sharing All Influenza Data (GISAID; https://gisaid.org, accessed May 29, 2025). Although considered part of the GMS, no sequence data were available from Myanmar. A/H3N2 3C.2a1b.2a.2a and subclade sequences isolated from human sources from all countries with collection dates between January 1, 2020, and December 31, 2023, and complete metadata fields for country of collection were downloaded from GISAID (accessed September 18, 2025).

### 2.7 Phylogenetic Analyses

Dated phylogenies of influenza A/H3N2 virus, influenza B virus, *Betacoronavirus 1*, RSV‐A, and RSV‐B were inferred via Bayesian analysis (BEAST v1.10.4 [22]). Consensus genomes were constructed as described above for samples meeting the sequence‐quality thresholds. For A/H3N2 and influenza B, consensus sequences were generated for genome segment 4, which encodes hemagglutinin (HA). Sequences were aligned by multiple alignment using MAFFT v7.490 [23].

First, cohort‐only analyses were performed for each of four viruses (A/H3N2, RSV‐A, RSV‐B, and *Betacoronavirus 1*) with statistically supported temporal clusters comprising at least 10 specimens each. Phylogenies were inferred under an HKY nucleotide‐substitution model with gamma‐distributed among‐site rate variation approximated using four rate categories, a strict molecular clock, and an exponential‐growth coalescent tree prior. Under the strict‐clock model, all branches were assumed to evolve at a common substitution rate. Virus‐specific initial clock rates and lognormal priors were used. For A/H3N2, the mean (and initial) clock rate was 0.004 substitutions/site/year, and the 95% prior clock rate interval (henceforth defined as the 2.5% and 97.5% quantiles of the prior distribution) was between 0.00169 and 0.00805 subs/site/year. For RSV‐A and RSV‐B, the mean (and initial) clock rate was 0.00175 substitutions/site/year, and the 95% prior clock rate interval was between 0.000738 and 0.00354 substitutions/site/year [24, 25]. For *Betacoronavirus 1*, the mean (and initial) clock rate was 0.0005 substitutions/site/year, and the 95% prior clock rate interval was between 0.000211 and 0.00101 substitutions/site/year [26, 27]. All other priors were left at their BEAST defaults.

Next, to place the cohort influenza sequences in contemporaneous phylogenetic context, additional analyses incorporated publicly available sequences collected in Cambodia, Laos, Thailand, and Vietnam (collectively: the GMS) during the same influenza season (2022). Cohort A/H3N2 and influenza B virus sequences were combined with sequences from human infections deposited in GISAID (total sequences referenced: 512 A/H3N2, 179 influenza B virus). A/H3N2 was modeled using the same substitution, site, clock, and exponential‐growth coalescent models as the corresponding cohort‐only analyses. For influenza B, the mean (and initial) clock rate was 0.0015 substitutions/site/year, and the 95% prior clock rate interval was between 0.000387 and 0.00406 substitutions/site/year. Because including all available contextual A/H3N2 sequences from the GMS would have substantially increased computational cost with limited expected benefit for interpretation, the available public sequences were subsampled before being combined with cohort sequences for the BEAST analyses. Public sequences were subsampled in two stages. First, pairwise genetic distances were calculated between the cohort sequences and the available public sequences, and each cohort sequence was then matched to a unique public sequence using the Hungarian assignment algorithm, which identified the set of one‐to‐one matches that minimized the total genetic distance across all matched pairs. To provide broader regional context beyond these genetically similar sequences, additional sequences were selected at random from the remaining public sequences. The final dataset therefore contained all cohort sequences, an equal number of optimally matched public sequences, and an additional random sample of public sequences. No subsampling was performed for the influenza B virus analysis.

Two additional analyses were conducted to examine phylogenetic patterns of A/H3N2 and influenza B viruses over a multi‐year period. For these, sequences collected across multiple years were analyzed using a Bayesian Skygrid coalescent model [28] rather than the exponential‐growth coalescent model. The Skygrid models effective population size as piecewise constant over a set of consecutive time intervals, allowing changes in effective population size to be estimated without imposing a single growth trajectory. The substitution, site‐rate, and molecular‐clock models remained otherwise unchanged, with the corresponding virus‐specific clock‐rate prior used for each analysis. In the first longitudinal analysis, cohort A/H3N2 and influenza B virus HA sequences were analyzed together with publicly available Cambodian HA sequences from matching clades collected between 2019 and 2023 (total sequences referenced: 181 A/H3N2, 84 influenza B virus). No subsampling was performed. In the second longitudinal analysis, the cohort sequences were combined with a geographically stratified subset of publicly available global A/H3N2 (clade 3C.2a1b.2a.2a) HA sequences collected between 2020 and 2023 (total sequences referenced = 43,444). Global sequences were stratified by World Health Organization (WHO) region and subsampled within each stratum. Within the Western Pacific Region, sequences were further stratified by country, with additional sampling from GMS countries to strengthen representation of viruses circulating in the subregion. The Skygrid model was parameterized with 50 effective‐population‐size intervals distributed across a 10‐year period. Nextstrain's Augur toolkit [29] was used to generate a starting tree for Markov chain Monte Carlo in BEAST.

BEAST analyses used one Markov chain of 20 million iterations each, logging every 1000 iterations. Convergence and mixing were evaluated by visual inspection of trace plots, and all monitored parameters had effective sample sizes greater than 200. Full model specifications can be found in the Github repository (URL: https://github.com/jessalynnsebastian/npseq_cambodia). Plots were generated and annotated using Tracer v1.7.2 [30], FigTree v1.4.4 [31], and R v4.3.1 using ape [32], tracerer [33], and phytools [34] packages.

## 3 Results

### 3.1 Diversity of Respiratory Viruses in Participants With Influenza‐Like Illness

**FIGURE 1:**
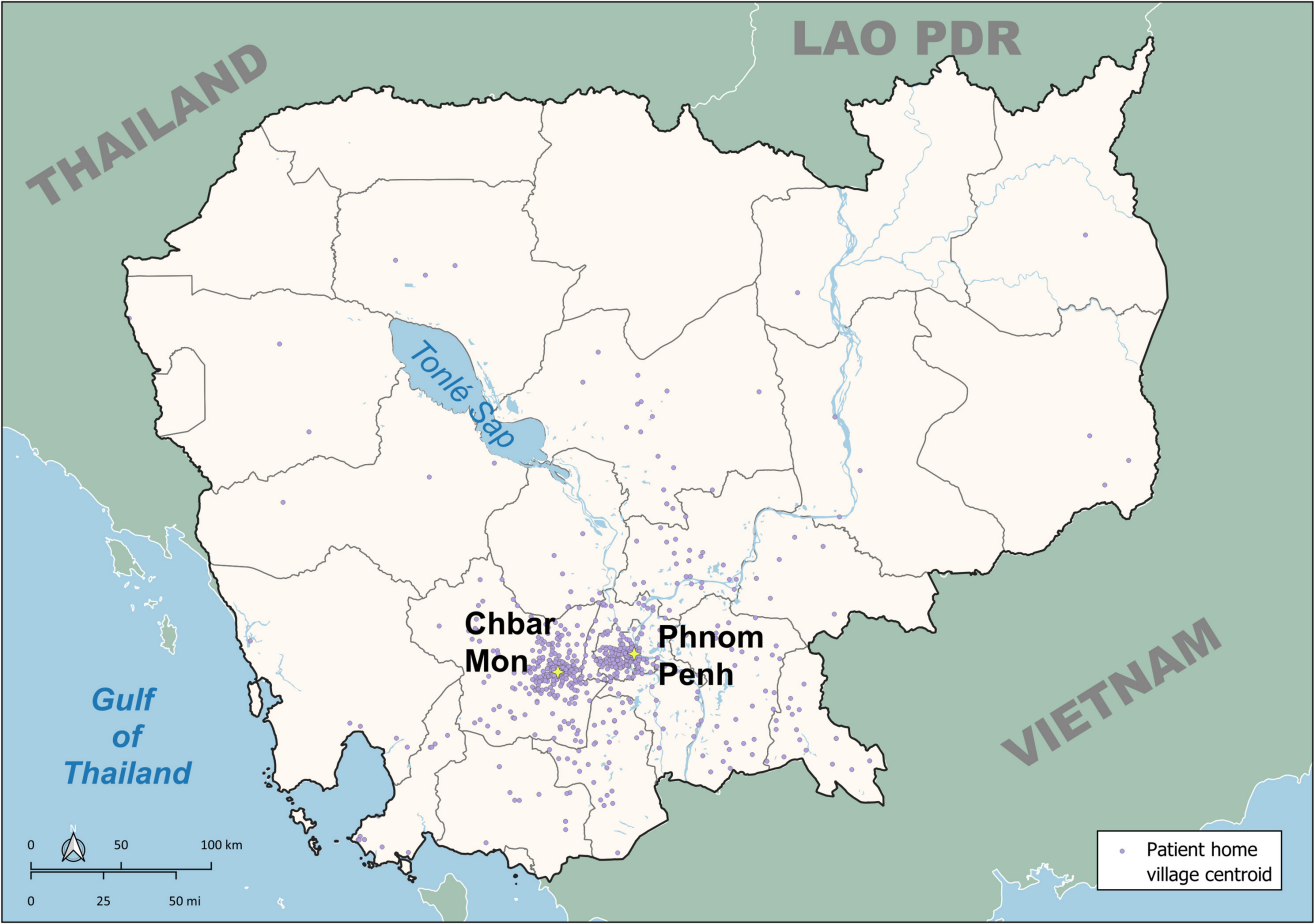
Geographical map of Cambodia showing locations (village centroids) of 1093 study participants with influenza‐like illness. Purple circles denote individual participants. Yellow stars and accompanying text labels indicate cities in which enrolling sites were located.

**FIGURE 2:**
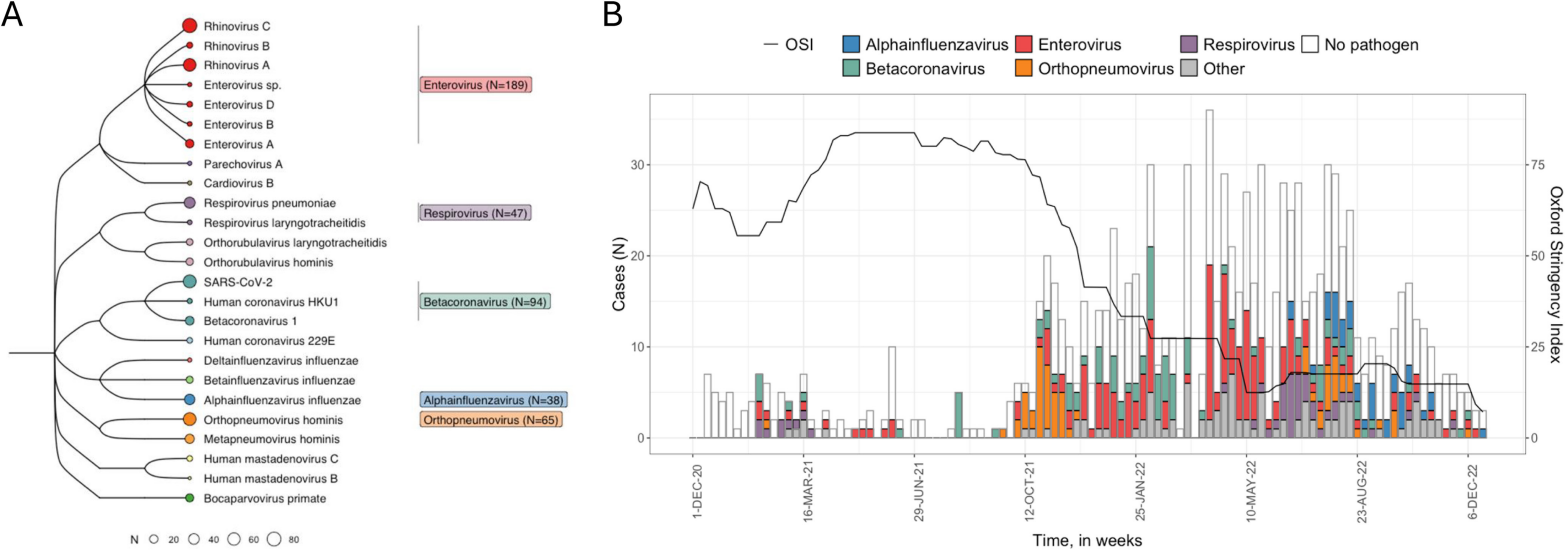
(A) Taxon cladogram of 533 respiratory viruses detected among 1093 participants with influenza‐like illness. Circle color and size indicate genus and number of samples, respectively. Text labels indicate the top five virus genera detected in the cohort. (B) Influenza‐like illness case counts over time (December 2020–March 2023). Stacked bars represent case counts colored by virus detected (left *y*‐axis). Solid black line represents lockdown measure stringency as indicated by the Oxford Stringency Index (OSI; right *y*‐axis). OSI data were obtained from the Global Change Data Lab (https://covid.ourworldindata.org/, accessed June 7, 2024).

From December 2020 to March 2023, 1093 individuals with ILI were enrolled across four hospitals (Table 1, Figure 1). The study population was mostly pediatric (median age 2 years, IQR 1–13) with low frequency of comorbid disease (underlying respiratory disease: 20/1093 [1.8%]; diabetes mellitus: 25/1093 [2.3%]; cardiac disease: 4/1093 [0.4%]; other comorbid disease: 50/1093 [4.6%]). A total of 533 respiratory viruses, including both DNA and RNA viruses, representing 68 distinct species were detected in 499 individuals corresponding to a case positivity rate of 45.7% (Tables S3–S4, Figure 2A). The most frequently detected genera were *Enterovirus* (189/533; 35.5%), *Betacoronavirus* (94/533; 17.6%), *Orthopneumovirus* (65/533; 12.2%), *Respirovirus* (47/533; 8.8%), and *Alphainfluenzavirus* (38/533; 7.1%). Co‐detection of > 1 virus was found among 34 individuals (3.1%) (Table S5). Respiratory viruses were not detected in 594 individuals (55.1%); these participants tended to be older (median age 9 years, IQR 1–30) than the overall cohort.

**TABLE 1:** Demographic characteristics of 1093 study participants with influenza‐like illness overall and stratified by major pathogen group.

Characteristic	Total, *N* = 1093	IAV, *N* = 38	BCoV, *N* = 94	EV, *N* = 189	OPV, *N* = 65	RPV, *N* = 47	Other, *N* = 100	No pathogen, *N* = 594
Age in years, median (IQR)	2 (1, 13)	11 (1, 20)	2 (1, 10)	1 (1, 2)	2 (1, 3)	1 (1, 3)	1 (1, 3)	9 (1, 30)
Female sex, *N* (%)	483 (44%)	13 (34%)	47 (50%)	90 (48%)	27 (42%)	19 (40%)	44 (44%)	254 (43%)
Occupation, *N* (%)								
Factory worker	44 (12%)	6 (29%)	2 (8.7%)	2 (10%)	0 (0%)	2 (40%)	2 (11%)	32 (12%)
Farmer	72 (20%)	1 (4.8%)	2 (8.7%)	2 (10%)	1 (25%)	0 (0%)	2 (11%)	64 (23%)
Forest worker	1 (0.3%)	0 (0%)	0 (0%)	0 (0%)	0 (0%)	0 (0%)	0 (0%)	1 (0.4%)
Military	3 (0.8%)	0 (0%)	0 (0%)	0 (0%)	0 (0%)	1 (20%)	0 (0%)	2 (0.7%)
Other	40 (11%)	2 (9.5%)	4 (17%)	3 (15%)	0 (0%)	0 (0%)	4 (21%)	28 (10%)
Police officer	1 (0.3%)	0 (0%)	0 (0%)	0 (0%)	0 (0%)	0 (0%)	0 (0%)	1 (0.4%)
Restaurant worker	1 (0.3%)	0 (0%)	0 (0%)	0 (0%)	0 (0%)	0 (0%)	0 (0%)	1 (0.4%)
School teacher	4 (1.1%)	0 (0%)	1 (4.3%)	1 (5.0%)	0 (0%)	0 (0%)	0 (0%)	2 (0.7%)
Student	187 (51%)	11 (52%)	12 (52%)	12 (60%)	3 (75%)	2 (40%)	10 (53%)	137 (49%)
Vendor	14 (3.8%)	1 (4.8%)	2 (8.7%)	0 (0%)	0 (0%)	0 (0%)	1 (5.3%)	10 (3.6%)
Unknown	726	17	71	169	61	42	81	316
Chronic lung disease	20 (1.8%)	1 (2.6%)	1 (1.1%)	5 (2.6%)	1 (1.5%)	0 (0%)	1 (1.0%)	12 (2.0%)
Chronic cardiac disease	4 (0.4%)	1 (2.6%)	0 (0%)	0 (0%)	0 (0%)	0 (0%)	1 (1.0%)	2 (0.3%)
Diabetes	25 (2.3%)	0 (0%)	0 (0%)	1 (0.5%)	0 (0%)	2 (4.3%)	0 (0%)	22 (3.7%)
Other comorbidity	50 (4.6%)	1 (2.6%)	2 (2.1%)	4 (2.1%)	1 (1.5%)	2 (4.3%)	3 (3.0%)	38 (6.4%)
Domestic travel within 30 days	46 (4.2%)	1 (2.6%)	11 (12%)	12 (6.3%)	5 (7.7%)	1 (2.1%)	1 (1.0%)	16 (2.7%)
International travel within 30 days	2 (0.2%)	0 (0%)	1 (1.1%)	0 (0%)	0 (0%)	0 (0%)	0 (0%)	1 (0.2%)
Site								
Kampong Speu District Referral Hospital	461 (42%)	18 (47%)	37 (39%)	60 (32%)	13 (20%)	6 (13%)	28 (28%)	305 (51%)
Preah Kossomak Hospital	135 (12%)	4 (11%)	16 (17%)	5 (2.6%)	2 (3.1%)	3 (6.4%)	4 (4.0%)	104 (18%)
National Pediatric Hospital	487 (45%)	13 (34%)	41 (44%)	123 (65%)	50 (77%)	37 (79%)	68 (68%)	180 (30%)
National Maternal and Child Health Center	10 (0.9%)	3 (7.9%)	0 (0%)	1 (0.5%)	0 (0%)	1 (2.1%)	0 (0%)	5 (0.8%)

Abbreviations: BCoV, betacoronavirus; EV, enterovirus; IAV, alphainfluenzavirus; IQR, interquartile range; OPV, orthopneumovirus; RPV, respirovirus.

### 3.2 Temporal Clustering of Respiratory Virus Cases

ILI cases were inversely associated with pandemic lockdown measure stringency (*p* < 0.001; Figure 2B, Table S6, Figures S1–S2). Case numbers averaged 12.5/month during lockdown (December 2020–October 2021) with a positivity rate of 40.1% (55/137). Among these, there were 8 SARS‐CoV‐2 versus 53 non‐SARS‐CoV‐2 cases (1:6.6), with a virus co‐detection rate of 10.9% (6/55). Nine PIV3 cases were detected between February and March 2021, though this cluster was not statistically supported. Following the gradual lifting of lockdown measures in October 2021, a significant temporal cluster of RSV‐A cases was identified (37 observed versus 5.6 expected cases; *p* < 0.0001; Table S7 and Figure S3). From November 2021, ILI cases rose to 56.2 cases/month with case positivity rate 46.4% (444/956). Among these, there were 59 SARS‐CoV‐2 and 413 non‐SARS‐CoV‐2 cases (1:7), with 6.3% (28/444) virus co‐detections. Enteroviruses and SARS‐CoV‐2 were among the first viruses to emerge in large numbers, though discrete temporal clusters were not found. Clusters of *Betacoronavirus 1* (*Human Coronavirus OC43*; 21 observed versus 6.9 expected cases; *p* < 0.0001) and *Orthorubulavirus hominis* (*Human Parainfluenzavirus 4*; 14 observed versus 6.1 expected cases; *p* = 0.0098) were identified between October 2021 and March 2022, and January 2022 to June 2022, respectively. Of note, there were no influenza cases detected in this cohort during the 2021 season. In mid‐2022, temporal clusters of RSV‐B (22 observed versus 4.5 expected cases; *p* < 0.0001), Influenza A/H3N2 virus (30 versus 9.5 expected cases; *p* < 0.0001), and Influenza B virus (12 versus 1.2 expected cases; *p* < 0.0001) occurred in rapid succession (Table S7, Figure S2). Among the six temporal viral clusters identified, smaller, spatiotemporal clusters were identified for RSV‐A, *Betacoronavirus 1*, *Orthorubulavirus hominis*, RSV‐B, A/H3N2, and influenza B virus, but not *Orthorubulavirus hominis* (Table S7).

### 3.3 Phylogeographic Investigation of Influenza A/H3N2, *Betacoronavirus 1*, and RSV Clusters

**FIGURE 3:**
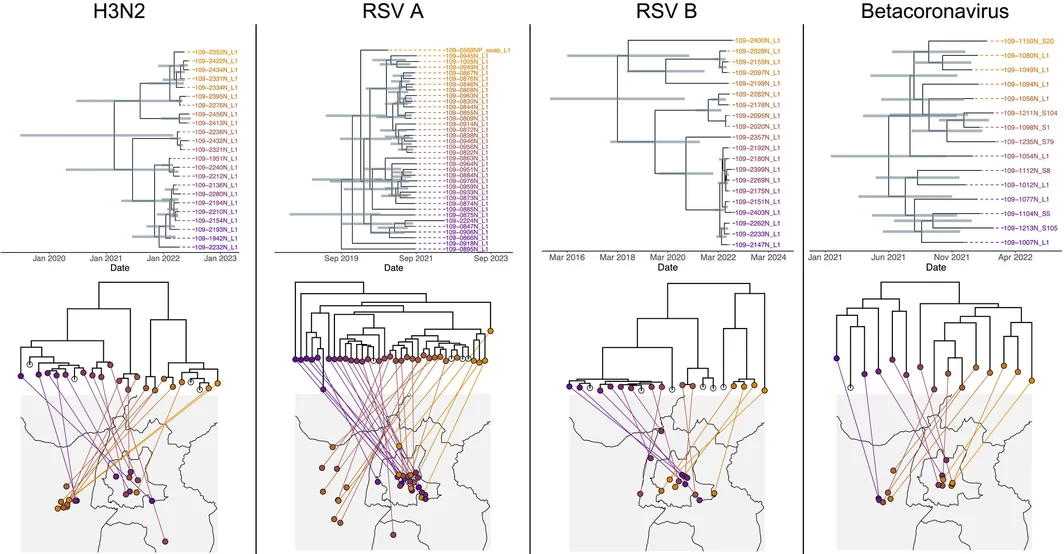
Maximum clade credibility time‐trees of influenza A/H3N2 virus, RSV‐A, RSV‐B, and *Betacoronavirus 1* full genome (or segment HA, for A/H3N2) sequences collected (top) mapped to geographic locations in Cambodia (bottom). Branch lengths represent time to most recent common ancestor for each clade. Phylogeny tips representing individual participant samples are connected by dotted lines to study participants' home village. Samples from participants living outside Phnom Penh and immediate surrounding provinces are indicated on the virus genealogies by empty circles but not on the corresponding maps.

To investigate potential transmission networks, dated viral phylogenies were constructed for virus species with at least 10 consensus genomes. All A/H3N2 strains belonged to clade 3C.2a1b.2a.2a. Overall posterior median clock rate of the HA segment was 0.0046 (95% highest posterior density [HPD] interval 0.0013–0.010) substitutions/site/year. Posterior median tree height, i.e., time to most recent common ancestor (tMRCA), was 1.31 (95% HPD 0.60–3.20) years and posterior median tree length was 6.86 (95% HPD 3.00–17.63) years. The geospatial distribution of cases across cluster epicenters in Phnom Penh and Kampong Speu did not mirror virus genealogy, i.e., viruses that were more closely related genetically were not necessarily from locations in close geographic proximity (Figure 3). Similarly, while pairwise geographic and time distances, and genetic and time distances, were weakly correlated (Mantel *r* 0.24, *p* = 0.023, and Mantel *r* 0.29, *p* = 0.0027, respectively; Figure S4), pairwise genetic and geographic distances were not (Mantel *r* 0.014, *p* = 0.40).

Relative to A/H3N2, RSV‐A and RSV‐B sequences had lower posterior median clock rates (0.00068 substitutions/site/year [95% HPD 0.00035–0.0011] and 0.0015 substitutions/site/year [95% HPD 0.00072–0.0024], respectively) and longer tMRCA (2.75 years [95% HPD 1.82–4.26] and 4.79 years [95% HPD 2.39–7.74], respectively) and posterior median tree length (24.72 years [95% HPD 14.47–43.28] and 25.20 years [95% HPD 12.35–40.70]. respectively). *Betacoronavirus 1* had a posterior median clock rate of 0.00034 substitutions/site/year (95% HPD 0.00016–0.00054) and the shortest tMRCA and posterior median tree length of all four viruses (0.71 years [95% HPD 0.38–1.10] and 5.96 years [95% HPD 2.83–9.86], respectively). As with A/H3N2, pairwise geographic and time distances for *Betacoronavirus 1* cases were weakly correlated (Mantel *r* 0.24, *p* = 0.036), but genetic and geographic distances, and genetic and time distances, were not (Mantel *r* 0.018, *p* = 0.42, and Mantel *r* − 0.039, *p* = 0.59, respectively; Figure S4), suggesting widespread physical distribution of genetically similar strains. For RSV‐A and RSV‐B, geographic, time, and genetic distances were not significantly correlated (Figure S4).

### 3.4 Local, Regional, and Global Influenza Epidemiology, 2020–2022

Globally, influenza A and B cases were almost completely absent in the first half of 2020 before picking up again a year and a half later in late 2021 (Figure S5). However, GMS countries reported sporadic cases of influenza A in late 2020 to early 2021, followed by a delayed period of quiescence for both influenza A and B through mid‐2022. The subsequent rise in influenza A cases in GMS countries lagged the corresponding seasons in the greater WHO Western Pacific Region (WPR; case reports dominated by China [56%] and Australia [17%]) and Southeast Asia Region (SEAR; case reports dominated by India [55%] and Nepal [18%]) by several months.

**FIGURE 4:**
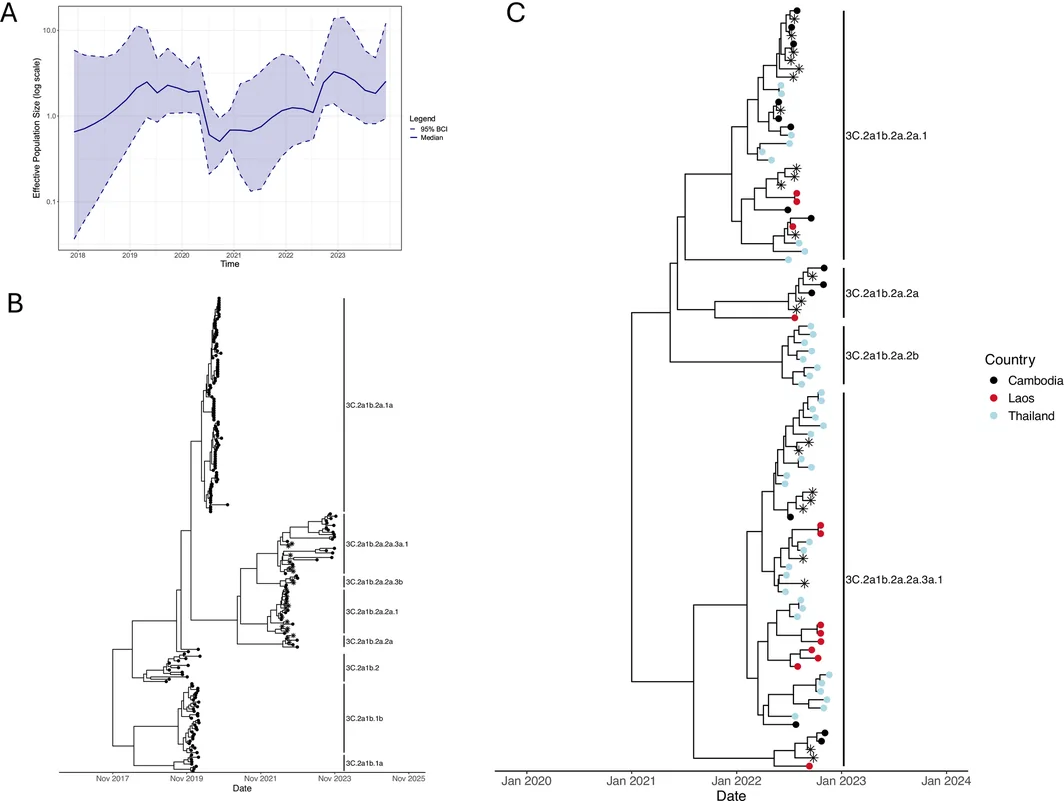
(A) Effective population size of influenza A/H3N2 based on HA segments from Cambodian isolates collected between 2019 and 2023. Solid and dotted lines indicate median and 95% Bayesian credible intervals, respectively. (B) Maximum clade credibility time‐tree of A/H3N2 based on HA segments from Cambodian isolates collected between 2019 and 2023. Asterisks represent sequences from this cohort. Black circles represent sequences downloaded from the Global Initiative on Sharing All Influenza Data (GISAID). Shaded grey rectangles indicate 95% Bayesian credible intervals. Subclades are indicated by brackets and text labels. (C) Maximum clade credibility time‐tree of A/H3N2 based on HA segments from isolates collected in Cambodia, Laos, and Thailand in 2022 (no sequences were available from Vietnam). Asterisks represent sequences from this cohort. Circles representing individual sequences are colored by country of isolate origin.

Among available Cambodian A/H3N2 HA sequences, strain diversity declined during 2020, with a slight recovery at the end of the year coinciding with the small rise in reported cases. Diversity increased further during the latter half of 2022, approaching levels observed before the pandemic (Figure 4A). Cohort A/H3N2 strains isolated during this resurgence diverged from those isolated prior to 2021 and intermixed with other contemporary Cambodian and GMS sequences (Figure 4B,C). Within global A/H3N2 3C.2a1b.2a.2a strains, Cambodian sequences formed eight distinct clusters that were interspersed among sequences from all six represented WHO regions but most closely associated with other sequences from WPR region countries (Figure S6).

Similar epidemiological and phylogenetic patterns were noted for influenza B: Both WPR and SEAR reported large influenza B seasons in late 2021 to early 2022 (majority of cases [81%] reported in China), despite minimal concurrent cases among GMS countries. GMS cases picked up later, coinciding instead with concurrent influenza seasons in the European (EUR) and Americas (AMR) regions in late 2022. Influenza B strain diversity in Cambodia decreased slightly from 2020 to 2021 and recovered in 2022 (Figure S7A). All eight full influenza B genomes recovered from this study between October and November 2022 were of B/Victoria lineage clade 1A.3a.2. These were distinct from historic Cambodian sequences, which belonged to clade 1A.3, and intermixed with Thai Influenza B sequences isolated in 2022 (Figure S7B–C). No Yamagata lineage isolates were detected in this cohort.

## 4 Discussion

Much of our understanding of respiratory virus genomic epidemiology originates from countries in the global North [35]. The democratization of sequencing technologies, accelerated in part by response to the COVID‐19 pandemic, has enabled LMICs to generate genomic surveillance data in‐country [36], increasing direct public health relevance of these data. In this study, we performed sub‐national surveillance of influenza‐like illness in Cambodia over 3 years and found circulation of diverse respiratory viruses particularly with easing of pandemic lockdown measures in late 2022. Although sampling was insufficient to study in‐country transmission networks, divergence of re‐emerging influenza A and B viruses from historic strains and rapid recovery of modest population diversity suggested multiple de novo introductions following the COVID‐19 pandemic. Influenza sequences inter‐mixed with other isolates in regional and global phylogenies but were genetically closest to contemporary sequences from other Western Pacific countries, suggesting enhanced transmission within this WHO region.

Cambodia experienced a relatively quiescent first year of the pandemic with only sporadic transmissions of SARS‐CoV‐2 [37]. It was not until February 2021 that the Cambodian Ministry of Health declared the country's first major community outbreak following a superspreader event [16]. Mobility restrictions were quickly escalated in response to burgeoning cases, culminating in full district‐level immobilizations between April and May 2021 [38]. However, SARS‐CoV‐2 continued to spread unabated with cases peaking at over 1000 per day during the Delta variant epidemic in June 2021 [16]. During this period of lockdown, we detected non‐SARS‐CoV‐2 respiratory viruses exceeding SARS‐CoV‐2 cases by over 6‐fold alongside high virus co‐detection rates (10.9%) in our sub‐national cohort. We suspect that asynchronous implementation and easing of lockdown measures across administrative levels and continued movement within districts despite inter‐province travel restrictions [38] may have sustained virus transmission at low levels. The sole exception were influenza viruses, which were notably absent during the 2021 rainy season mirroring trends reported elsewhere [3]. The near‐global “disappearance” of influenza reveals its reliance on circulation between countries and hemispheres to sustain continued transmission and evolution. In contrast, the robust endemic cycles of rhino/enteroviruses, RSV, and other common respiratory viruses may be sufficient to support mid‐ to long‐term survival of these viruses within smaller host pools.

On November 1, 2021, stringency measures in Cambodia were lifted in the context of widespread vaccination and the country was declared “reopened” [38]. Resurgence of respiratory viruses in our cohort during this period was brisk and extensive with ILI cases increasing by over 4‐fold. Both SARS‐CoV‐2 and non‐SARS‐CoV‐2 cases rose, the former dominated by the highly transmissible Omicron variant and the latter initially by diverse enteroviruses. Other respiratory viruses followed soon after, notably RSV and influenza during the 2022 rainy season, the latter which had been conspicuously absent the prior year. We hypothesized that genetic bottlenecks may have occurred during reduced circulation and sought to detect these with resurgences post‐lockdown. However, enterovirus, RSV, and PIV3 populations within our cohort were already surprisingly diverse by 2022, and we had no means to compare these measures to baseline levels in the absence of pre‐pandemic data. We were able to identify largely monophyletic re‐emergences of influenza A and B in our cohort (A/H3N2/3C.2a1b.2a.2a and B/Victoria/1A.3a.2, respectively) and compare these against historic and contemporary genomes in public repositories. We found that Cambodian H3N2 strains in 2022 diverged from pre‐pandemic strains in Cambodia and appeared to lag homologous seasons elsewhere. Taken together, these findings could suggest external introduction events or at the very least, extensive local evolution that was relatively shielded from global transmission networks.

In Cambodia, a LMIC in the WHO Western Pacific Region (WPR), granularity of ILI data is limited due to inclusion of fewer than a dozen sentinel surveillance sites [13, 14]. On the other hand, high‐income countries such as the United States, China, and Australia tend to generate the bulk of GISRS data from which guidance on preparedness planning and vaccine strain selection is derived [3]. While it is beneficial for LMICs lacking robust in‐country surveillance infrastructure to receive such guidance, the data skew could mean that public health policies are not tailored to a country's specific needs. Our data from this limited study suggest that influenza epidemiology in Cambodia may not follow that within the WPR, which in 2022 was largely driven by reporting from China and Australia. Instead, the timing and genetic composition of Cambodia's post‐pandemic influenza re‐emergence may have more closely followed that of neighbors in the GMS. This is perhaps an unsurprising observation given the high degree of human movement between these countries [39], along with shared climatology, both of which are critical drivers of respiratory virus seasonality and epidemiology [40]. Similarly, others have reported SARS‐CoV‐2 importation events over land borders from Thailand in the north and Vietnam to the south [16]. The region's interconnectedness highlights the relevance of developing regional partnerships that could enable resource pooling, enhance data generation, and facilitate concerted action to tackle local disease priorities.

Our study has several limitations. Cases were sampled at four tertiary hospitals, which, while covering a large catchment area, were not nationally representative. Study design involved active case finding at participating hospitals but no contact tracing or community surveillance. Importantly, screening procedures and recruitment intensity remained unchanged throughout the study period. However, the apparent decrease in virus circulation during the height of pandemic‐associated lockdown may have been due to changes in care‐seeking behavior or other confounders, rather than decreases in transmission per se. The limited retrospective analysis presented here assessed the association between lockdown stringency and ILI cases and should not be interpreted as establishing a causal relationship. Severity and clinical outcomes data were not collected as part of the approved study protocol, limiting inferences on viral virulence. Further, viral presence alone does not confirm disease; it is possible that ILI in enrolled cases may not have been caused by the virus detected. Our metagenomic workflow only screened for known viruses with published genomes in public repositories; emerging viruses and variants may have been missed. Finally, limited availability of public Influenza virus sequences from Cambodia precluded more detailed phylogeographic analyses and limited inferences regarding number, timing, and source of viral introductions.

The COVID‐19 pandemic had significant impacts on respiratory virus epidemiology, while simultaneously forcing growth in countries' capacity to detect, report, and respond to respiratory virus outbreaks. In our sub‐national surveillance network, metatranscriptomic sequencing detected and characterized diverse viruses associated with ILI between 2020 and 2023. The unique timing and strain composition of influenza re‐emergence in 2022 suggest that external introductions may have contributed to virus population diversity in Cambodia.

## Author Contributions

**Christina Yek:** conceptualization, investigation, writing – original draft, methodology, validation, visualization, data curation, supervision, resources, project administration. **Jessalyn Sebastian:** investigation, writing – original draft, software, formal analysis, data curation, visualization. **Sophana Chea:** investigation, writing – review and editing, methodology, data curation, supervision. **Sreyngim Lay:** investigation, methodology, writing – review and editing, data curation. **Mengheng Oum:** investigation, methodology, writing – review and editing, data curation. **Lyhourng Long:** investigation, methodology, writing – review and editing, data curation. **Sreytouch Chea:** investigation, methodology, writing – review and editing, data curation. **Andrea R. Pacheco:** investigation, methodology, writing – review and editing, data curation, visualization. **Meera Barochia:** investigation, methodology, writing – review and editing, data curation. **Piseth Ly:** investigation, methodology, writing – review and editing, data curation. **Sokna Ly:** investigation, methodology, writing – review and editing, data curation. **Ratanak Sath:** investigation, methodology, writing – review and editing, data curation. **Daniel M. Parker:** methodology, software, visualization, formal analysis, writing – review and editing. **Volodymyr M. Minin:** methodology, writing – review and editing, software, formal analysis, supervision. **Matthew Chung:** methodology, writing – review and editing, software, formal analysis. **Elodie Ghedin:** methodology, writing – review and editing, software, formal analysis, supervision. **Fabiano Oliveira:** investigation, methodology, writing – review and editing, supervision, project administration. **Jessica E. Manning:** investigation, methodology, writing – review and editing, funding acquisition, project administration, supervision. **Kimsreng Lean:** investigation, methodology, writing – review and editing, resources. **Chanty Ny:** investigation, methodology, writing – review and editing, resources. **Viseth Long:** investigation, methodology, writing – review and editing, resources. **Kimhor Leang:** investigation, methodology, writing – review and editing, resources. **Virak Yim:** investigation, methodology, writing – review and editing, resources. **Kry** **Hok:** investigation, methodology, writing – review and editing, resources. **Rithea Leang:** investigation, methodology, writing – review and editing, project administration. **Rekol Huy:** investigation, methodology, writing – review and editing, project administration. **Savuth Chin:** investigation, methodology, writing – review and editing, project administration. **Darapheak Chau:** investigation, methodology, writing – review and editing, project administration. **Heng Seng:** investigation, methodology, writing – review and editing, project administration, resources. **Sovann Ly:** investigation, methodology, writing – review and editing, project administration, resources. **Chanthap Lon:** conceptualization, investigation, writing – original draft, methodology, validation, project administration, supervision, data curation, resources.

## Funding

This research was supported in part by the Intramural Research Program of the National Institutes of Health (NIH) and the Bill and Melinda Gates Foundation [grant number OPP1211806]. The contributions of the NIH authors are considered Works of the United States Government. The findings and conclusions presented in this paper are those of the author(s) and do not necessarily reflect the views of the NIH or the US Department of Health and Human Services. The funding source was not involved in study design, data collection, data analysis, data interpretation, or preparation of this report.

## Ethics Statement

The study protocol was approved by the National Institutes of Health Institutional Review Board and the Cambodian National Ethics Committee for Human Research and registered on clinicaltrials.gov (NCT04034264).

## Consent

Written informed consent was obtained prior to study enrollment from all research participants or their legal guardian(s).

## Conflicts of Interest

JEM became an employee of Sanofi Inc. after completion of her work for this study.

## Supporting information


**Figure S1:** Influenza‐like illness case counts over time (December 2020—March 2023) by (A) participant age group and (B) study site. Stacked bars represent case counts (left *y*‐axis). Solid black line represents lockdown measure stringency as indicated by the Oxford Stringency Index (OSI; right *y*‐axis).
**Figure S2:** Associations between monthly study enrollment, hospital admissions, and the Oxford Stringency Index (OSI). (A) Monthly study enrollment period plotted against total monthly hospital admissions. (B) Monthly study enrollment period plotted against OSI. (C) Predicted monthly study enrollment as a function of the OSI based on the fitted negative binomial regression model. Points represent monthly observations and blue lines represent fitted linear regression models (A,B). Black solid line represents the model‐predicted monthly enrollment (C). Shaded regions denote the 95% confidence interval (A,B,C).
**Figure S3:** Temporal clusters of RSV‐A, Betacoronavirus 1, orthorubulavirus hominis, RSV‐B, A/H3N2, and influenza B virus. Filled blue bars represent the number of detected cases per month. Dark gray shaded regions indicate time periods corresponding to statistically significant temporal clusters identified by SaTScan.
**Figure S4:** Scatter plots of unique pairwise viral genetic distance (number of small nucleotide polymorphisms [SNPs]) versus geographic distance (kilometers) versus time distance (number of days) between individual cases of Betacoronavirus 1, A/H3N2, RSV‐A, and RSV‐B. Each point represents a single pairwise comparison. Correlation between distance matrices was assessed using Mantel tests. Spearman Mantel correlation coefficient (r) and permutation p values are reported within each panel.
**Figure S5:** Cases of influenza A (A,B) and influenza B (C,D) reported to the World Health Organization (WHO) Global Influenza Surveillance and Response System between January 1, 2020, and December 31, 2022. Global cases (A,C) are shaded by WHO region (AFR, African Region; AMR, Region of the Americas; EMR, Eastern Mediterranean Region; EUR, European Region; SEAR, Southeast Asia Region; WPR, Western Pacific Region). Cases in the Greater Mekong Subregion (GMS) are shaded by country.
**Figure S6:** Maximum clade credibility tree of cohort and publicly available A/H3N2 HA segments belonging to clade 3C.2a1b.2a.2a. Asterisks represent sequences from this cohort. Circles representing individual sequences are colored by country of WHO region (legend).
**Figure S7:** (A) Effective population size of influenza B virus based on HA segments from Cambodian isolates collected between 2019 and 2023. Solid and dotted lines indicate median and 95% Bayesian credible intervals, respectively. (B) Maximum clade credibility time‐tree of influenza B based on HA segments from Cambodian isolates collected between 2019 and 2023. Asterisks represent sequences from this cohort. Black circles represent sequences downloaded from the Global Initiative on Sharing All Influenza Data (GISAID). Subclades are indicated by brackets and text labels. (C) Maximum clade credibility time‐tree of influenza B based on HA segments from isolates collected in Cambodia and Thailand in 2022 (no sequences were available from Laos or Vietnam). Asterisks represent sequences from this cohort. Circles representing individual sequences are colored by country of isolate origin.

**Table S1:** Demographic, clinical, and virological data of 1093 participants with influenza‐like illness.
**Table S2:** Consensus sequences for virus genomes or segments with greater or equal to 90% genome identity and 10X coverage breadth.
**Table S3:** Taxonomy of 533 respiratory viruses detected in 1093 study participants with influenza‐like illness.
**Table S4:** Cohort characteristics and pathogen hit, stratified by participant age and study site.
**Table S5:** Detected respiratory viruses among 34 participants with detection of > 1 virus.
**Table S6:** Negative binomial model parameters for ILI cases using OSI and monthly admissions as predictors.
**Table S7:** Temporal clusters of respiratory viruses identified by Bernoulli space‐time scan statistics.

## Data Availability

De‐identified individual participant data are available in the Supplement. All fastq were deposited in NCBI's Sequence Read Archive under Bioproject PRJNA681566. Source code for the phylogenetic analyses and data visualizations has been shared in a public repository (https://github.com/jessalynnsebastian/npseq_cambodia).
