# Supplementary material for: Mapping Sub‐National Respiratory Virus Circulation in Cambodia Using Metatranscriptomic Sequencing: A Multi‐Center Hospital‐Based Surveillance Study
Source: Influenza Other Respir Viruses. 2026 Aug 19;20(8):e70306. doi: 10.1111/irv.70306 (PMC13490045; doi:10.1111/irv.70306)
Supplement: Supplementary file 1 [file IRV-20-e70306-s002.pdf]

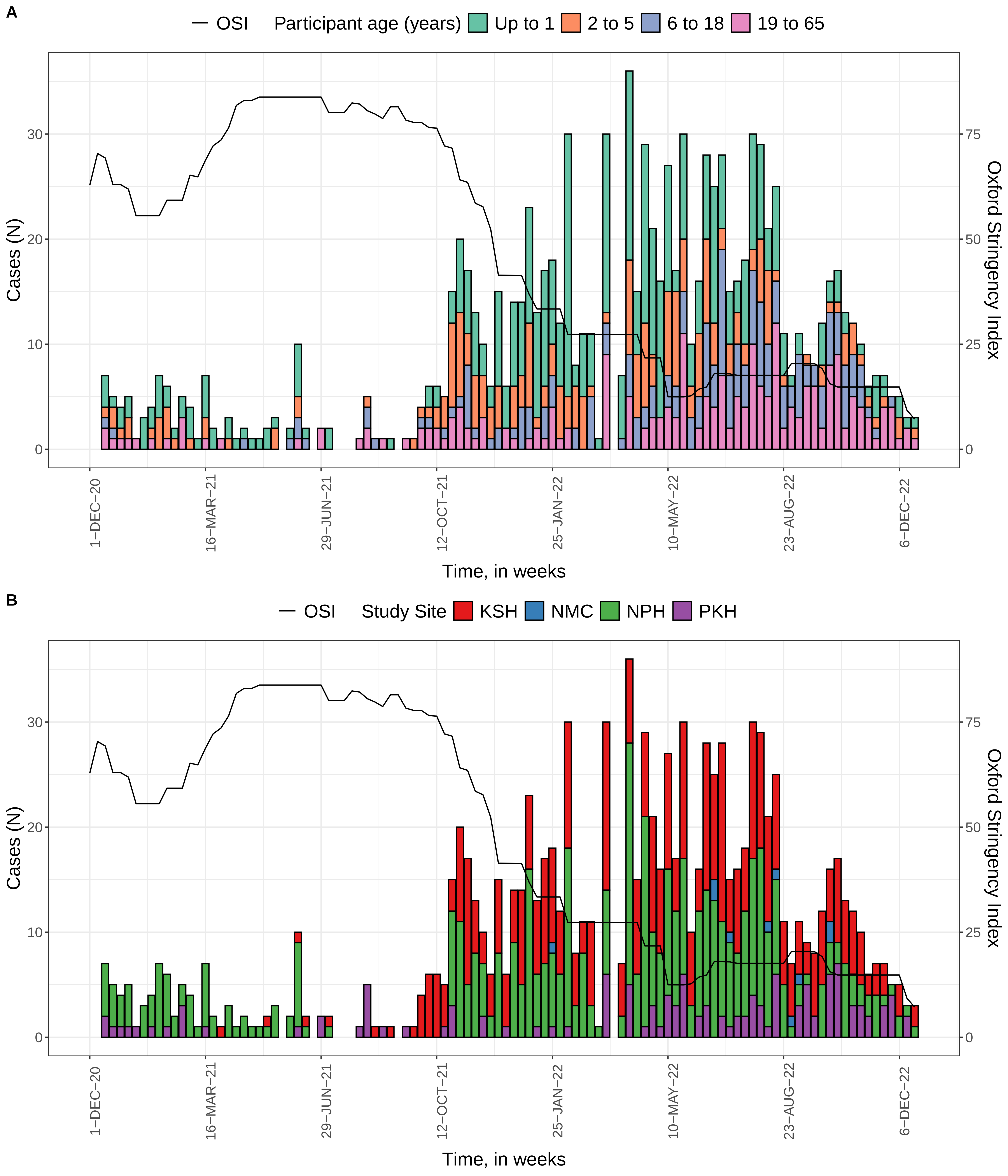
A
OSI
Participant age (years)
Up to 1
2 to 5
6 to 18
19 to 65
Cases (N)
Oxford Stringency Index
0
10
20
30
0
25
50
75
1−DEC−20
16−MAR−21
29−JUN−21
12−OCT−21
25−JAN−22
10−MAY−22
23−AUG−22
6−DEC−22
Time, in weeks
B
OSI
Study Site
KSH
NMC
NPH
PKH
Cases (N)
Oxford Stringency Index
0
10
20
30
0
25
50
75
1−DEC−20
16−MAR−21
29−JUN−21
12−OCT−21
25−JAN−22
10−MAY−22
23−AUG−22
6−DEC−22
Time, in weeks

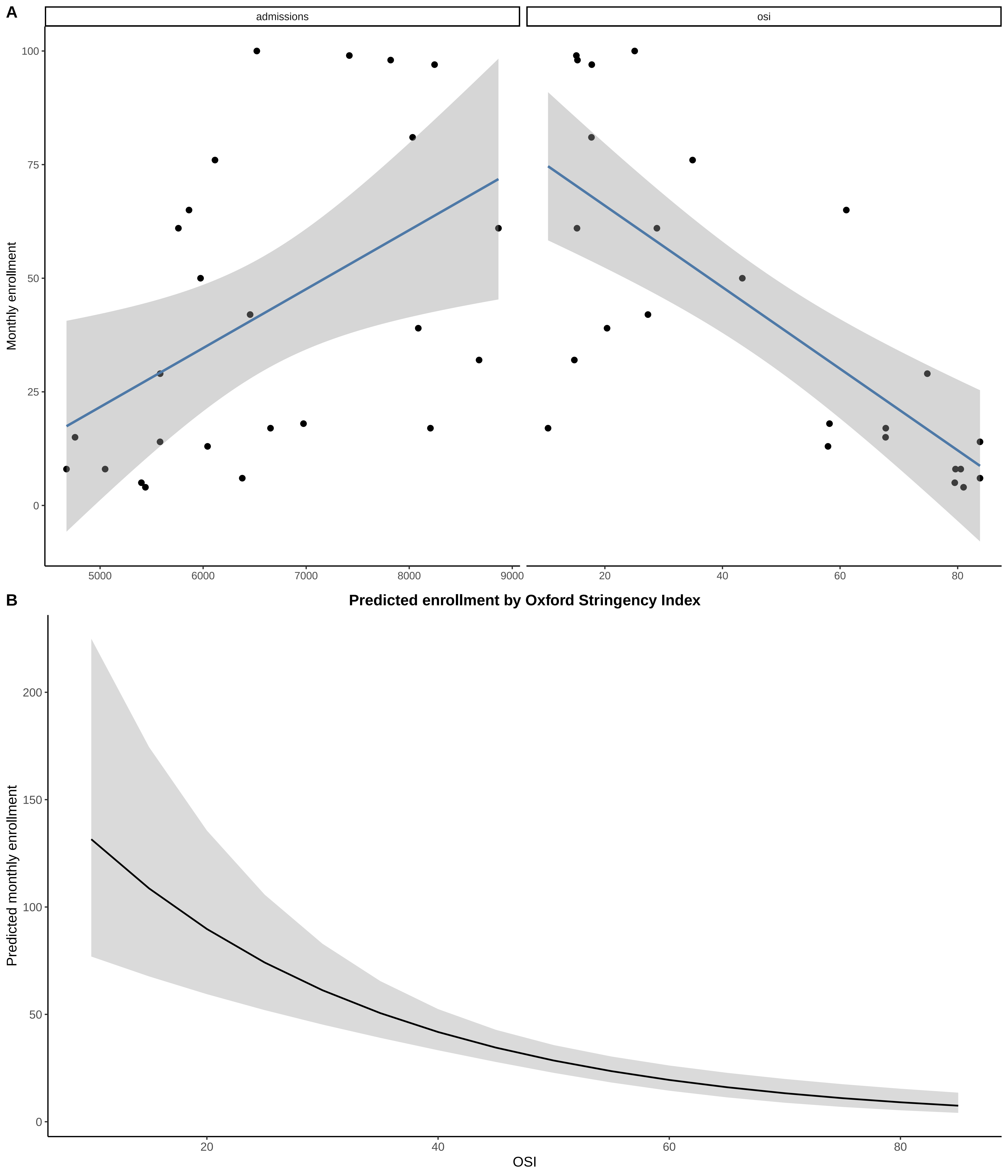
A
admissions
osi
Monthly enrollment
100
75
50
25
0
5000
6000
7000
8000
9000
20
40
60
80
B
Predicted enrollment by Oxford Stringency Index
Predicted monthly enrollment
200
150
100
50
0
20
40
60
80
OSI

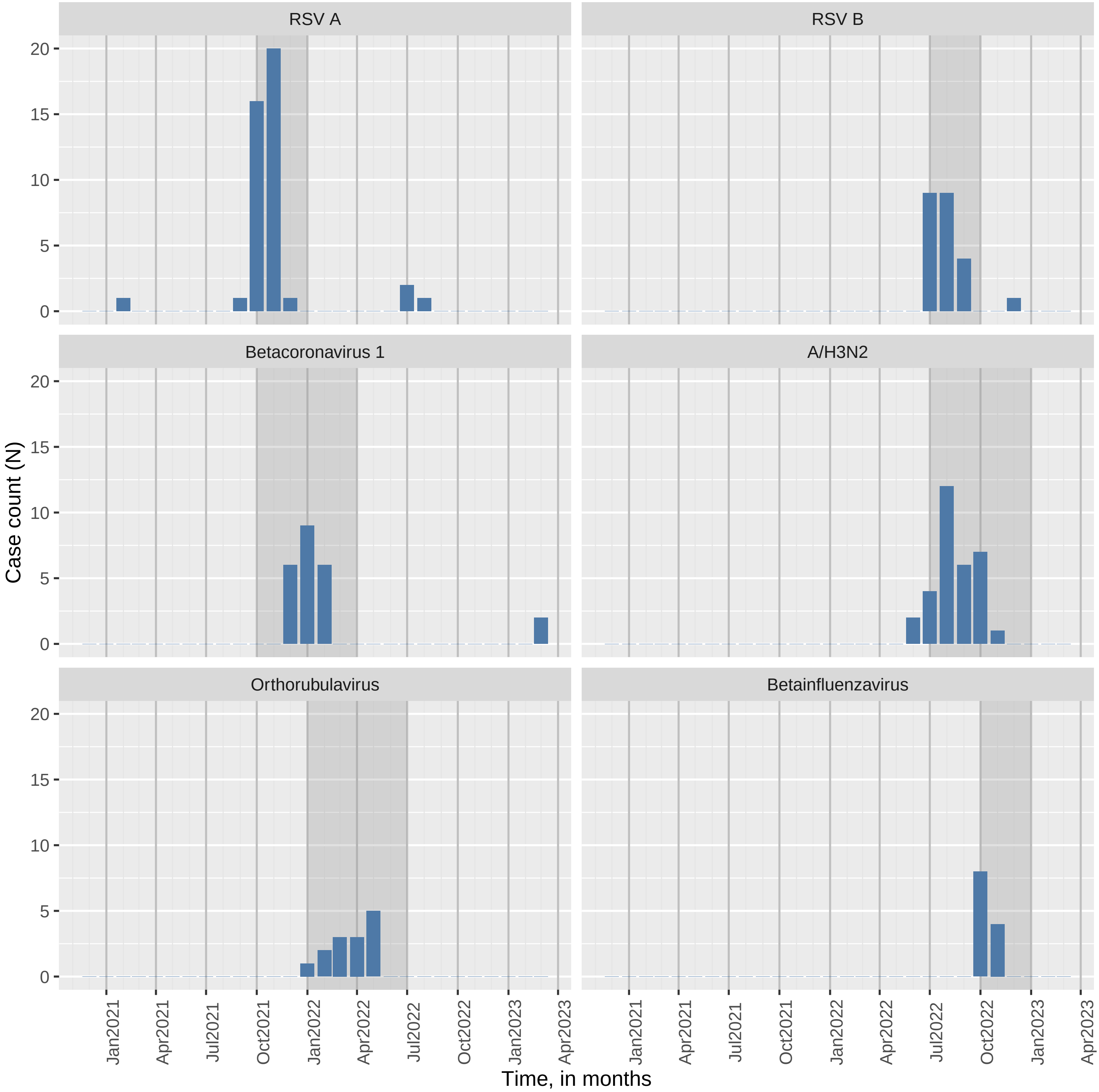

RSV A
RSV B
Betacoronavirus 1
A/H3N2
Orthorubulavirus
Betainfluenzavirus
Case count (N)
0
5
10
15
20
Jan2021
Apr2021
Jul2021
Oct2021
Jan2022
Apr2022
Jul2022
Oct2022
Jan2023
Apr2023
Time, in months

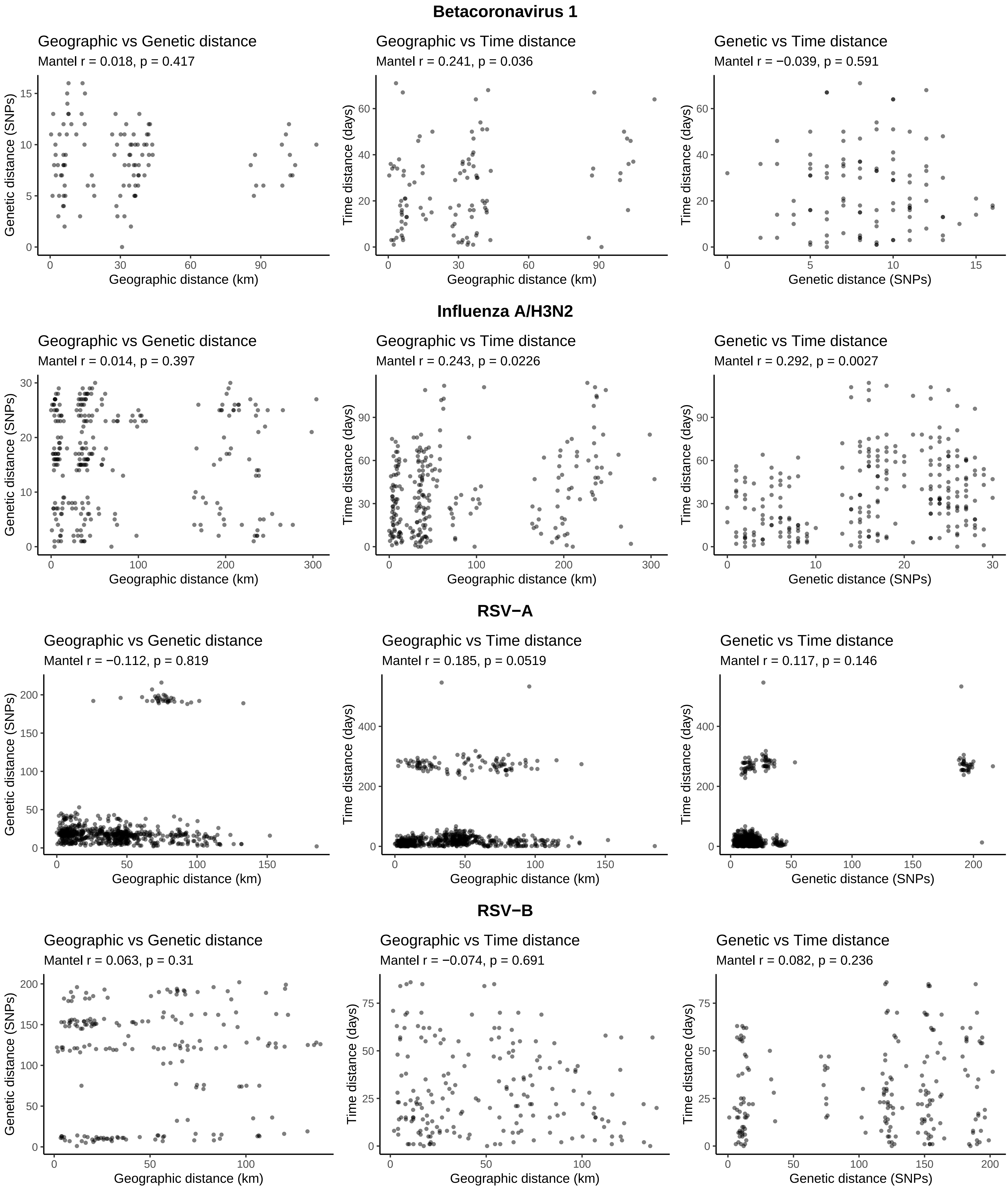

Betacoronavirus 1
Geographic vs Genetic distance
Mantel r = 0.018, p = 0.417
Genetic distance (SNPs)
Geographic distance (km)
Geographic vs Time distance
Mantel r = 0.241, p = 0.036
Time distance (days)
Geographic distance (km)
Genetic vs Time distance
Mantel r = −0.039, p = 0.591
Time distance (days)
Genetic distance (SNPs)
Influenza A/H3N2
Geographic vs Genetic distance
Mantel r = 0.014, p = 0.397
Genetic distance (SNPs)
Geographic distance (km)
Geographic vs Time distance
Mantel r = 0.243, p = 0.0226
Time distance (days)
Geographic distance (km)
Genetic vs Time distance
Mantel r = 0.292, p = 0.0027
Time distance (days)
Genetic distance (SNPs)
RSV−A
Geographic vs Genetic distance
Mantel r = −0.112, p = 0.819
Genetic distance (SNPs)
Geographic distance (km)
Geographic vs Time distance
Mantel r = 0.185, p = 0.0519
Time distance (days)
Geographic distance (km)
Genetic vs Time distance
Mantel r = 0.117, p = 0.146
Time distance (days)
Genetic distance (SNPs)
RSV−B
Geographic vs Genetic distance
Mantel r = 0.063, p = 0.31
Genetic distance (SNPs)
Geographic distance (km)
Geographic vs Time distance
Mantel r = −0.074, p = 0.691
Time distance (days)
Geographic distance (km)
Genetic vs Time distance
Mantel r = 0.082, p = 0.236
Time distance (days)
Genetic distance (SNPs)

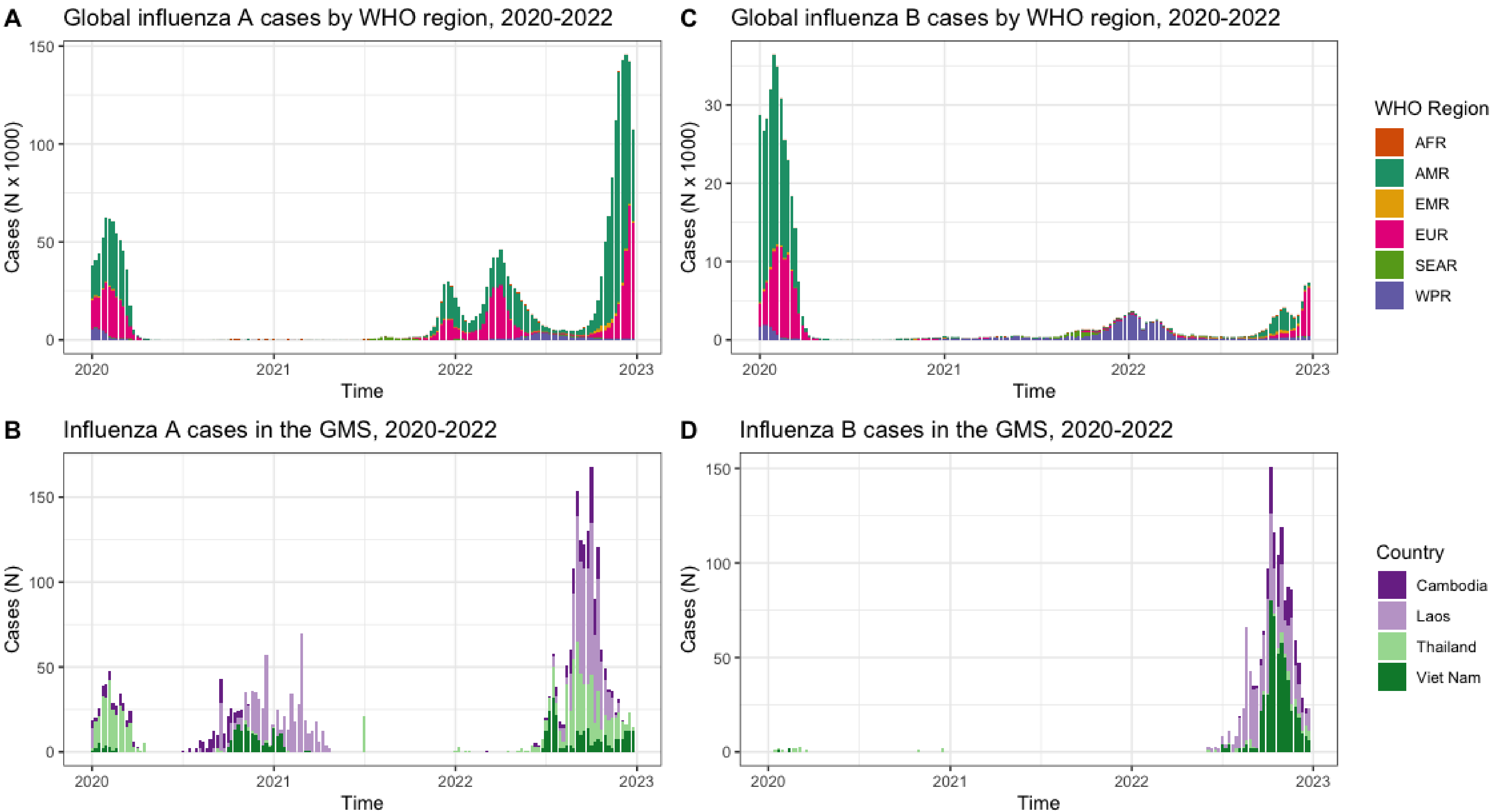

A
Global influenza A cases by WHO region, 2020-2022
Cases (N x 1000)
Time
B
Influenza A cases in the GMS, 2020-2022
Cases (N)
Time
C
Global influenza B cases by WHO region, 2020-2022
Cases (N x 1000)
Time
D
Influenza B cases in the GMS, 2020-2022
Cases (N)
Time
WHO Region
AFR
AMR
EMR
EUR
SEAR
WPR
Country
Cambodia
Laos
Thailand
Viet Nam

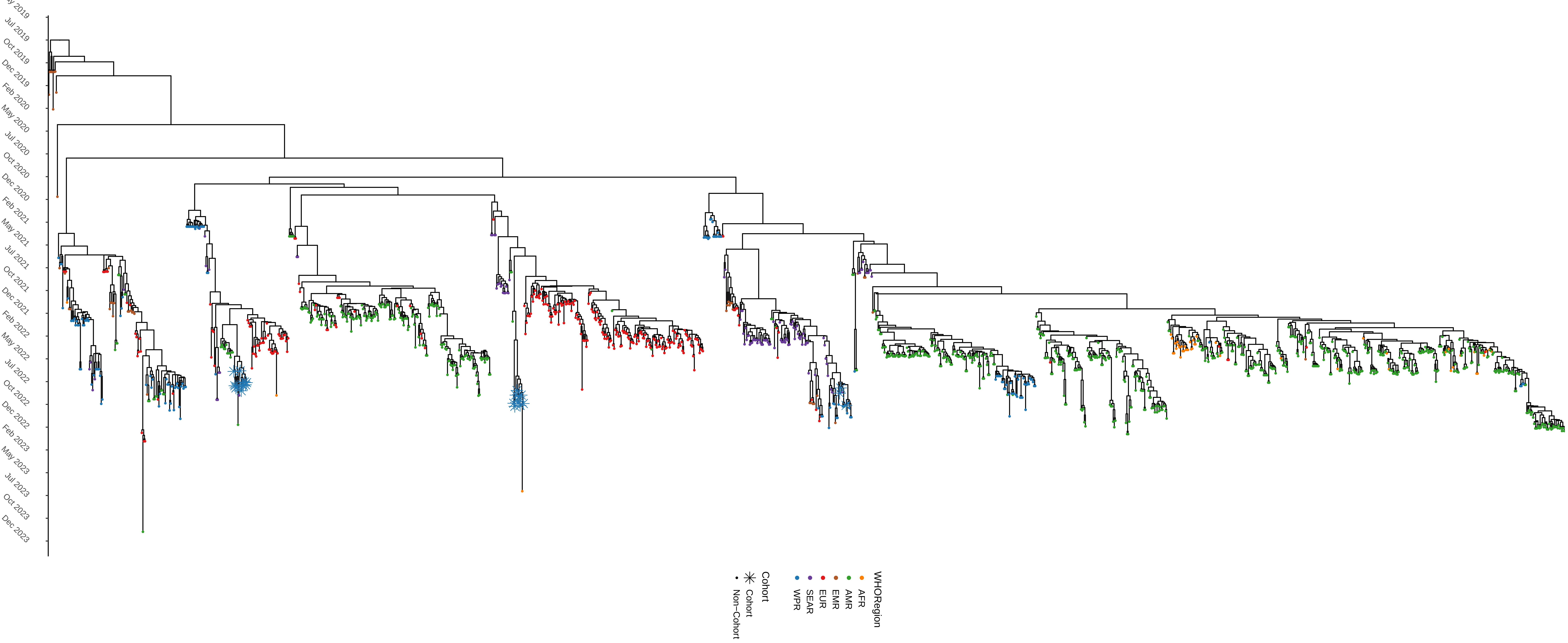

Jul 2019
Oct 2019
Dec 2019
Feb 2020
May 2020
Jul 2020
Oct 2020
Dec 2020
Feb 2021
May 2021
Jul 2021
Oct 2021
Dec 2021
Feb 2022
May 2022
Jul 2022
Oct 2022
Dec 2022
Feb 2023
May 2023
Jul 2023
Oct 2023
Dec 2023
Cohort
Cohort
Non−Cohort
WHORegion
AFR
AMR
EMR
EUR
SEAR
WPR

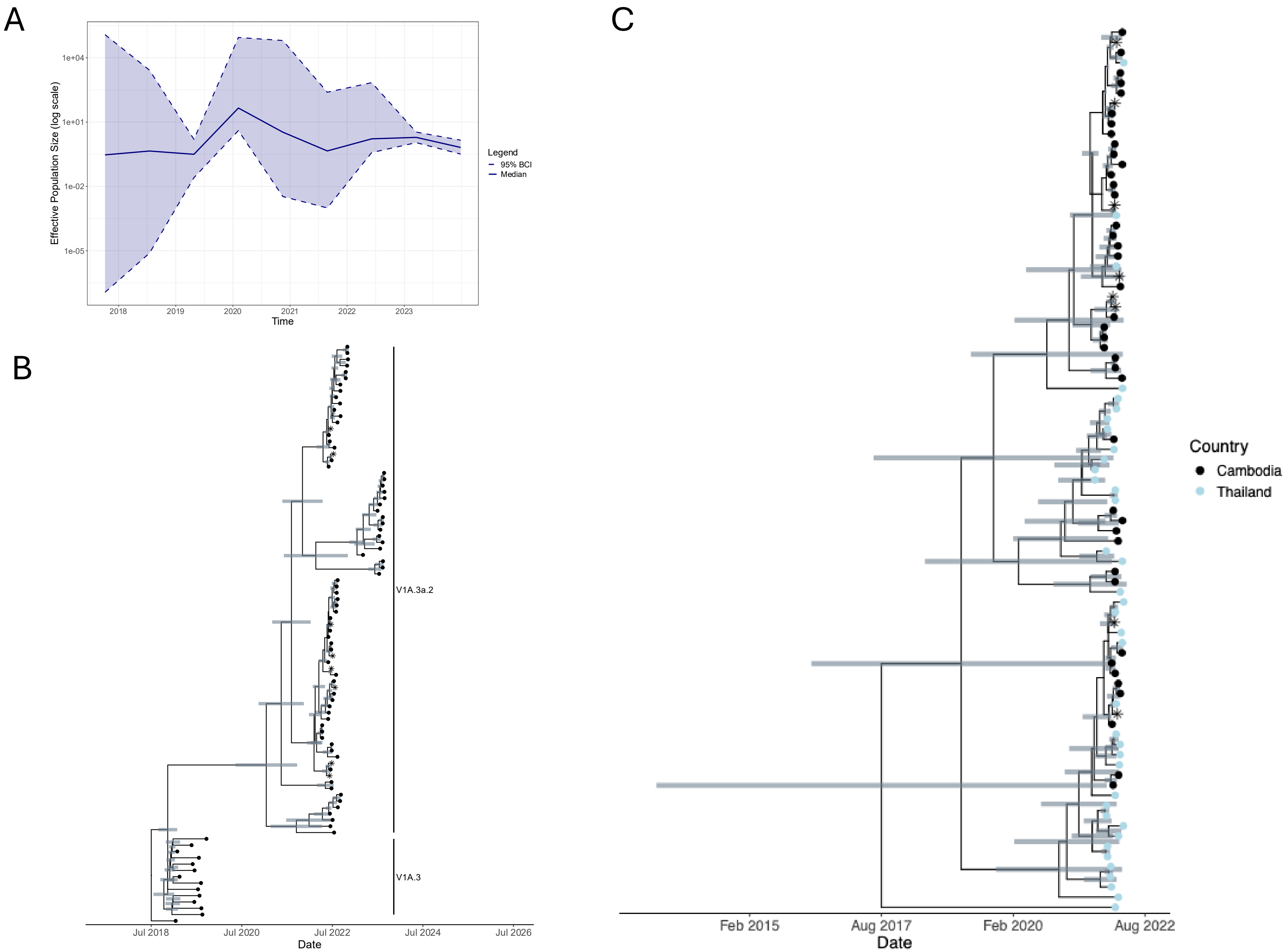
A
Effective Population Size (log scale)
1e+04
1e+01
1e-02
1e-05
2018
2019
2020
2021
2022
2023
Time
Legend
95% BCI
Median
B
V1A.3a.2
V1A.3
Jul 2018
Jul 2020
Jul 2022
Jul 2024
Jul 2026
Date
C
Country
Cambodia
Thailand
Feb 2015
Aug 2017
Feb 2020
Aug 2022
Date
